# Epidemiological and spatiotemporal analysis of elderly HIV-1/AIDS patients in Ningxia, China, from 2018 to 2023

**DOI:** 10.1038/s41598-025-98791-6

**Published:** 2025-04-23

**Authors:** Zhonglan Wu, Yichang Liu, Xiaofa Ma, Yufeng Li, Xiaohong Zhu, Dongzhi Yang, Jianxin Pei, Yong Li

**Affiliations:** 1 College of Life Sciences, Ningxia University, No.489, West Helanshan Road, Xixia District, Yinchuan, 750004 Ningxia Hui Autonomous Region China; 2 School of Public Health, Ningxia Medical University, Yinchuan, Ningxia Hui Autonomous Region China; 3 Ningxia Hui Autonomous Region Center for Disease Control and Prevention, NO.4 Fengchao Road, Yinchuan, 750004 Ningxia Hui Autonomous Region China

**Keywords:** Epidemiological analysis, Spatiotemporal analysis, HIV-1, Elderly population, HIV infections, Epidemiology

## Abstract

To analyze the epidemiological transmission characteristics and spatiotemporal distribution patterns of the elderly HIV-1/AIDS population in NHAR from 2018 to 2023, to provide theoretical support for the targeted formulation and implementation of HIV-1 interventions. A cross-sectional study was conducted in August 2024. Plasma samples were collected from the elderly HIV-1/AIDS patients (> 50 years old) in NHAR, followed by RNA extraction and RT-PCR to amplify the pol gene of HIV-1. The amplicons were sequenced for the partial pol region. Subtyping was performed using online tools from the HIV-1 database and MEGA11. Drug resistance was analyzed using the Stanford University HIVdb algorithm. Molecular transmission networks were constructed using Cytoscape 3.10.0. Logistic regression was performed to identify the potential risk factors. Spatial analysis revealed the geographic patterns of elderly HIV-1/AIDS patients. A total of 208 HIV-1/AIDS patients were included in this study, predominantly male (78.37%), primary school and below (46.63%), heterosexual transmission (80.77%) and farmers (52.40%). Nine genetic subtypes were identified, with CRF07_BC being the most common (54.81%). The overall drug resistance rate was 37.98%. The number of network nodes increased from 18 in 2018 to 107 in 2023, with large propagating clusters in 2023 merging or expanding from smaller clusters in previous years. Logistic regression analysis showed that males had a lower risk of transmission, individuals from Yinchuan, Shizuishan, and Wuzhong had a lower probability of entering the network, and CRF07_BC and CRF01_AE had a higher risk of transmission. From 2020 to 2023, there was a highly significant clustering pattern among elderly HIV/AIDS patients in NHAR, with shifts in hotspots. Yuanzhou District remained a persistent cold spot. This study reveals that the elderly HIV-1/AIDS patients in NHAR were predominantly married, male, and engaged in farming, with low levels of education. An increase in the diversity of viral genetic subtypes was observed, along with a high rate of drug resistance. The molecular network expanded significantly, accompanied by the emergence of large transmission clusters, indicating complex transmission patterns. The spatial distribution of these cases exhibited aggregation, with notable differences observed between districts and counties. To effectively intervene in the transmission of HIV-1 among the elderly population, it is essential to establish a long-term dynamic molecular transmission surveillance network and to improve AIDS screening and drug resistance testing.

## Introduction

AIDS is a chronic infectious disease caused by the human immunodeficiency virus (HIV). As of 2023, 39.9 million people globally were living with HIV^1^. Despite the progress made in HIV prevention and control efforts, the proportion of elderly HIV/AIDS patients among the total patient population is steadily rising due to the intensifying ageing of the population and improved survival rates associated with antiretroviral therapies (ART)^2^. In HIV/AIDS research, an age threshold of ≥ 50 years is commonly used to distinguish this group from the typically defined sexually active population (15–49 age group)^3,4^. Generally, HIV/AIDS patients aged 50 and above^5^, who are thus classified as elderly HIV/AIDS patients, pose a significant burden^6^. In 2020, 48.1% of all newly diagnosed HIV/AIDS cases in China were among people aged 50 and above^7^. Elderly HIV/AIDS patients confront a multitude of health crises. Deterioration of their immune system function leads to weakened resistance to pathogens, increasing the risk of co-infection and complications. Moreover, the frequent occurrence of HIV-related neurocognitive disorders, coupled with the increasing prevalence of age-related chronic noncommunicable diseases, further compounds the health burden of these patients^8^. The combined effect of these factors underscores the significance of elderly HIV/AIDS patients as a priority group in HIV/AIDS prevention and control efforts.

The Ningxia Hui Autonomous Region (NHAR), located in northwest China and bordering provinces such as Shaanxi and Gansu Provinces, has a highly mobile population. Despite notable advancements in HIV-1 prevention and control strategies, positioning it as a low-prevalence region within China, recent years have seen an alarming increase in the detection rate of drug resistance following ART. If the high rates of drug resistance are not promptly addressed, elderly HIV-1/AIDS patients face accelerated disease progression and increased susceptibility to opportunistic infections due to their compromised immune systems. Moreover, drug resistant strains are more likely to circulate within elderly populations and potentially spread to other vulnerable groups, leading to an expanded reservoir of drug-resistant virus carriers. Notably, existing studies in NHAR predominantly focus on the general population, while systematic spatiotemporal epidemiological analyses targeting elderly HIV-1/AIDS patients remain absent. This oversight neglects age-specific risk factors such as resumption of sexual behaviour after widowhood, lack of age-appropriate health education, potential failure to identify high-risk transmission clusters, and spatial disparities within this population. This lack of comprehensive data further complicates prevention and control efforts, highlighting the urgent need for targeted studies that can inform effective strategies for this vulnerable demographic. This study aims to analyze the characteristics of subtype distribution, degree of drug resistance, and molecular transmission network of HIV-1/AIDS among the elderly in NHAR, as well as the spatial distribution and clustering of elderly HIV-1/AIDS patients at the county or district level from 2018 to 2023, providing valuable information for the implementation of targeted interventions.

## Methods

### Study participants

A cross-sectional study was conducted in August 2024, utilizing blood samples from elderly HIV-1/AIDS patients in Ningxia collected between 2018 and 2023 from the biorepository. Inclusion criteria for this study were as follows: (1) participants had to be HIV-1/AIDS patients residing in NHAR; (2) aged 50 years or older; (3) included both HIV-1/AIDS patients on ART and those not on ART; (4) had received a viral load test between January 2018 and December 2023 with a viral load ≥ 400 copies/ml; (5) demographic information could be correctly matched with laboratory data; and (6) all participants had given informed consent to participate in the survey. On the other hand, the exclusion criteria included: (1) samples with failed HIV nucleic acid extraction, amplification, or sequencing; (2) duplicate sequences from the same HIV-1/AIDS patient; (3) viral load < 400 copies/mL; and (4) samples with missing or incorrect demographic data.

### Laboratory tests

Blood plasma samples were collected and stored at -80℃ before RNA extraction. Viral RNA was extracted from plasma utilizing the automated nucleic acid extraction and purification system, along with the HIV-1 viral load assay according to the manufacturer’s protocol (Zhuhai Livzon Diagnostics Inc). Partial pol sequences corresponding to codons 1 to 99 aa of the protease and 1-299 aa of the reverse transcriptase were amplified by in-house nested reverse transcription PCR using the One Step RNA PCR Kit (Takara, China). The amplified products were analyzed by 1% agarose gel electrophoresis, and the positive products were selected for gene sequencing at SinoGenoMax Co., Ltd. (Beijing, China).

### Identification of HIV-1 genotypes

The genetic subtype was initially identified using the BLAST tool from the HIV-1 database (https://www.hiv.lanl.gov/). For inconclusive gene sequences, preliminary recombination analysis was conducted using the online tools RIP and jpHMM from the HIV-1 Database. The sequences were aligned and adjusted using Clustal W in MEGA 11 software. The most appropriate evolutionary model was determined based on Bayesian Information Criterion (BIC) scores. Finally, a phylogenetic tree was constructed using the Neighbor-Joining method with a Bootstrap value of 1000 to identify the HIV-1 genetic subtype and analyze its epidemiological characteristics.

### Genotypic resistance analysis

DRMs and resistance levels were determined based on the Stanford HIV db Program (https://hivdb.stanford.edu/hivdb/by-sequences/). The database categorizes DRMs into five levels: susceptible (S), potential resistance (P), low-level resistance (L), intermediate resistance (I), and high-level resistance (H). In this study, the presence of low-level or higher resistance to any drug was considered as genotypic resistance.

### Molecular transmission network and spatial analysis

Cytoscape 3.10.0 was used to construct the molecular transmission network based on a pairwise genetic distance (GD) under the Tamura-Nei (TN93) nucleotide substitution model. The pilot analysis was conducted with a range of pairwise genetic distances from 0.1 to 1.5%, incremented in 0.2% intervals, for comprehensive evaluation.

### General spatial autocorrelation

This study investigated the clustering of HIV/AIDS cases among the elderly population at the county level in NHAR using the Global Moran’s I index. The Global Moran’s I value ranges between − 1 and 1, with a Moran’s I > 0 and a z-score > 1.96, indicating a clustered distribution of infections. In contrast, the distribution is considered dispersed when Moran’s I < 0 and the z-score < -1.96, while other scenarios suggest a random distribution^9,10^.

### Local spatial autocorrelation

Local spatial autocorrelation analysis was used to examine the connections between AIDS case distributions within a county and its neighboring regions. The Getis-Ord Gi* statistical method was employed to assess the degree of local case clustering by calculating z-scores and P-values. Counties with z-scores higher than 1.96 were identified as hotspots, indicating a significant concentration of AIDS cases. In contrast, those with z-scores below − 1.96 were classified as cold spots, indicating a relatively sparse distribution of cases^11,12^. The entire analyses, including map generation, were performed using ArcGIS 10.8.1 software(ESRI, Inc., Redlands, CA, USA; https://www.esri.com/arcgis).

### Statistics analysis

Statistical analyses were performed using STATA V.17.0 (STATA, USA) and Excel 2010. Count data were expressed in frequencies. Chi-square or Fisher exact tests were employed to compare proportions between different groups. Univariate and multivariate logistic regression models were used to analyze potential factors associated with entering the network. All tests were two-tailed, and P values < 0.05 were considered statistically significant.

### Ethics declarations

The research protocols of the present study were approved by the Institutional Review Board of the Ningxia Hui Autonomous Region Center for Disease Control and Prevention (No.2024-LLSC-089). All methods were performed in accordance with the relevant guidelines and regulations.

## Results

### Participant characteristics and genotype analysis

A total of 312 elderly HIV/AIDS patients were enrolled in the study. Based on the inclusion criteria, a final cohort of 208 individuals was successfully sequenced and analyzed.

Among the participants, 78.37% (163/208) were male and 21.63% (45/208) were female. In terms of marital status, 67.31% (140/208) were married or partnered, 30.29% (63/208) were divorced or widowed, and 2.40% (5/208) were unmarried. Most participants had an education level of primary school or below (46.63%, 97/208), with a majority living in Xingqing District (12.98%). The predominant mode of transmission was heterosexual (80.77%), and 52.40% of the individuals identified as farmers (Table 1). The geographical distribution of elderly HIV-1 infected individuals in NHAR from 2018 to 2023 is shown in Table 2.

**Fig. 1:**
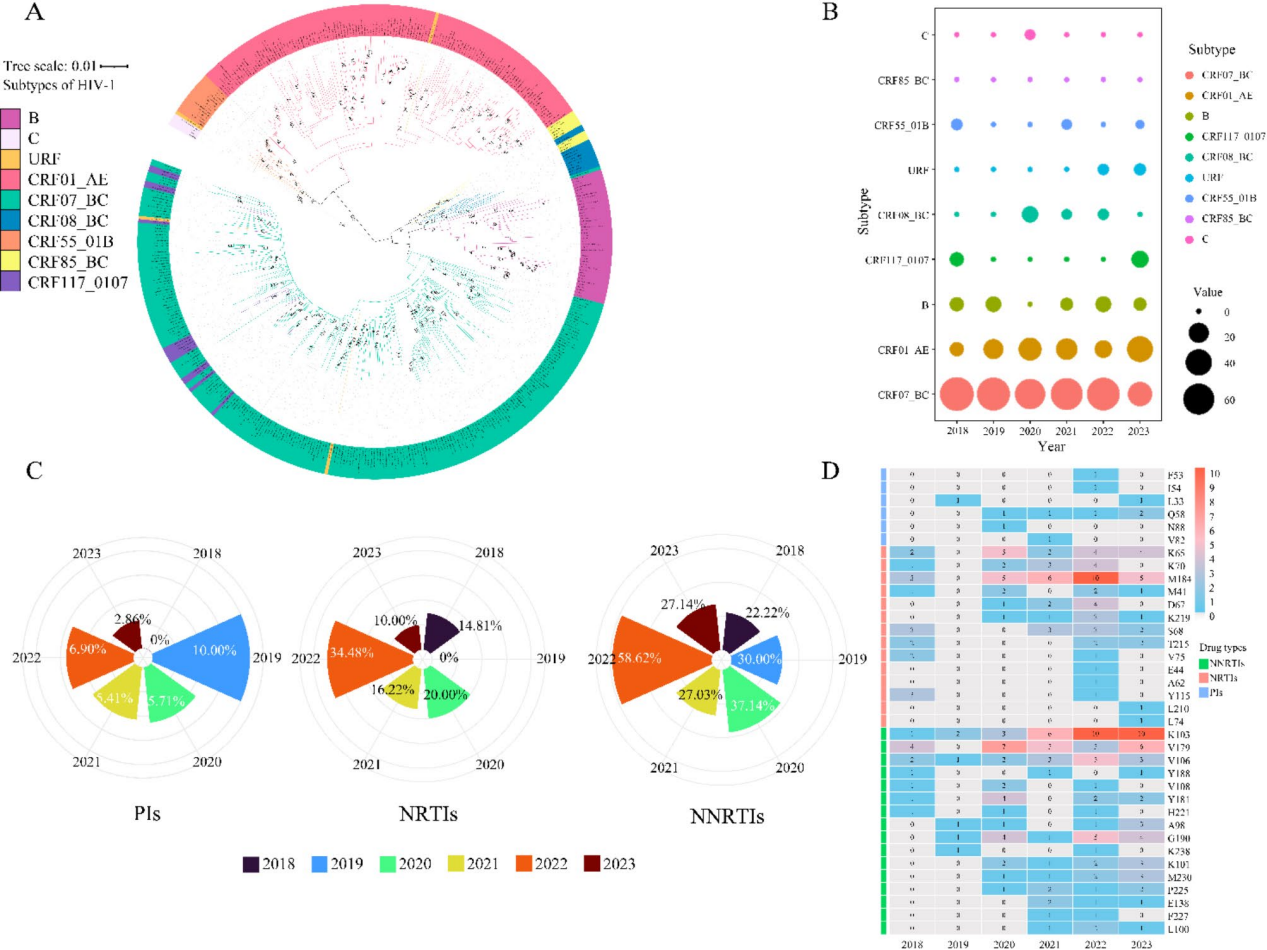
(**A**) Phylogenetic analysis of the pol sequences and gene subtypes of elderly HIV/AIDS patients in NHAR, China, 2018–2023. (**B**) The proportion dynamics of subtypes in NHAR from 2018 to 2023. (**C**) Dynamic changes in resistance rates of PIs, NRTIs, and NNRTIs in NHAR from 2018 to 2023. (**D**) Changes in the number of DRMs in elderly HIV/AIDS patients in NHAR from 2018 to 2023.

The most common genotype among all participants was CRF07_BC (54.81%, 114/208), followed by CRF01_AE (25.96%, 54/208). Other genotypes included subtype B (5.77%, 12/208), CRF117_0107 (5.30%, 11/208), CRF08_BC (2.88%, 6/208), unique recombinant forms (URFs) (1.92%, 4/208), CRF55_01B (1.44%, 3/208), CRF85_BC (1.44%, 3/208) and subtype C (0.48%, 1/208). The phylogenetic analysis of the system is shown in Fig. 1A. The HIV/AIDS subtypes statistically significant differences in onset time (*p* < 0.05) and drug resistance status (*p* < 0.05), with even more pronounced statistical differences in terms of network affiliation (*p* < 0.001) and place of residence (*p* < 0.001), as shown in Table 1. CRF07_BC emerged as the dominant subtype from 2018 to 2023, accounting for more than 50% of all cases annually. Notably, in 2023, there was a rapid surge in the prevalence of the CRF01_AE subtype, which surpassed other subtypes to attain the highest proportion (Fig. 1B).

**Table 1:** The demographic characteristics and subtype distribution of elderly HIV/AIDS patients in NHAR from 2018 to 2023.

Demographic classification		Total (*N* = 208)	CRF01_AE (*n* = 54)	CRF07_BC (*n* = 114)	Others^*^ (*n* = 40)	c^2^ value		*P* value
Age, (n/%)						6.294		0.614
50 ~ 54		70 (33.65)	12 (22.22)	41 (35.96)	17 (42.05)			
55 ~ 59		46 (22.12)	13 (24.07)	24 (21.05)	9 (22.50)			
60 ~ 64		34 (16.35)	11 (20.37)	19 (16.67)	4 (10.00)			
65 ~ 69		20 (9.62)	5 (9.26)	11 (9.65)	4 (10.00)			
70~		38 (18.27)	13 (24.07)	19 (16.67)	6 (15.00)			
Gender, (n/%)						1.190		0.552
Female		45 (21.63)	12 (22.22)	22 (19.30)	11 (27.50)			
Male		163 (78.37)	42 (77.78)	92 (80.70)	29 (72.50)			
Occupation, (n/%)						5.257		0.730
Unemployed/retiree		61 (29.33)	11 (20.37)	38 (33.33)	12 (30.00)			
Farmer		109 (52.40)	31 (57.41)	57 (50.00)	21 (52.50)			
Business waiter		9 (4.33)	2 (3.70)	6 (5.26)	1 (2.50)			
Workers		25 (12.02)	8 (14.81)	12 (10.53)	5 (12.50)			
Others and unknown		4 (1.92)	2 (3.70)	1 (0.88)	1 (2.50)			
Marital status, (n/%)						4.831		0.305
Married/Partnered		140 (67.31)	35 (64.81)	77 (67.54)	28 (70.00)			
Divorced/Widowed		63 (30.29)	19 (35.19)	32 (28.07)	12 (30.00)			
Unmarried		5 (2.40)	0 (0.00)	5 (4.39)	0 (0.00)			
Educational level, (n/%)						5.138		0.526
Primary school and Below		97 (46.63)	27 (50.00)	49 (42.98)	21 (52.50)			
Junior high school		74 (35.58)	19 (35.19)	40 (35.09)	15 (37.50)			
High school/Technical secondary school		26 (12.50)	6 (11.11)	16 (14.04)	4 (10.00)			
Junior college and above		11 (5.29)	2 (3.70)	9 (7.89)	0 (0.00)			
Routes of infection, (n/%)						7.176		0.127
Heterosexual transmission		168 (80.77)	48 (88.89)	89 (78.07)	31 (77.50)			
Homosexual transmission		37 (17.79)	6 (11.11)	24 (21.05)	7 (17.50)			
Drug injection		3 (1.44)	0 (0.00)	1 (0.88)	2 (5.00)			
Onset time, (n/%)						25.465		< 0.05
2018~		27 (12.98)	2 (3.70)	20 (17.54)	5 (12.50)			
2019~		10 (4.81)	2 (3.70)	7 (6.14)	1 (2.50)			
2020~		35 (16.83)	10 (18.52)	20 (17.54)	5 (12.50)			
2021~		37 (17.79)	9 (16.67)	24 (21.05)	4 (10.00)			
2022~		29 (13.94)	4 (7.41)	20 (17.54)	5 (12.50)			
2023~		70 (33.65)	27 (50.00)	23 (20.18)	20 (50.00)			
Drug resistance (n/%)						9.999		< 0.05
No		129 (62.02)	43 (79.63)	62 (54.39)	24 (60.00)			
Yes		79 (37.98)	11 (20.37)	52 (45.61)	16 (40.00)			
Enter network, (n/%)						18.609		< 0.001
No		101 (48.56)	15 (27.78)	57 (50.00)	29 (72.50)			
Yes		107 (51.44)	39 (72.22)	57 (50.00)	11 (27.50)			
Place of residence						25.413		< 0.001
Yinchuan City		106 (50.96)	25 (46.30)	59 (51.75)	22 (55.00)			
Shizuishan City		17 (8.17)	6 (11.11)	9 (7.89)	2 (5.00)			
Wuzhong City		44 (21.15)	7 (12.96)	28 (24.56)	9 (22.50)			
Guyuan City		20 (9.62)	2 (3.70)	12 (10.53)	6 (15.00)			
Zhongwei City		21 (10.10)	14 (25.93)	6 (5.26)	1 (2.50)			

Others^*^ including B、C、CRF55_01B、CRF117_0107、CRF08_BC、CRF85_BC and URF subtype.

**Table 2:** Geographical distribution of elderly HIV-infected Individuals in NHAR from 2018 to 2023.

Residence	Case/n (%)
Yinchuan City	
Xingqing District	27 (12.98)
Xixia District	20 (9.62)
Jinfeng District	16 (7.69)
Yongning County	12 (5.77)
Helan County	15 (7.21)
Lingwu City	16 (7.69)
Shizuishan City	
Dawukou District	5 (2.40)
Huinong District	4 (1.92)
Pingluo County	8 (3.85)
Wuzhong City	
Litong District	24 (11.54)
Hongsipu District	1 (0.48)
Yanchi County	11 (5.29)
Tongxin County	4 (1.92)
Qingtongxia City	4 (1.92)
Guyuan City	
Yuanzhou District	6 (2.88)
Xiji County	9 (4.33)
Longde County	2 (0.96)
Jingyuan County	2 (0.96)
Pengyang County	1 (0.48)
Zhongwei City	
Shapotou District	14 (6.73)
Zhongning County	4(1.92)
Haiyuan County	3 (1.44)

### Drug resistance analysis

The resistance rate for protease inhibitors (PIs) showed a significant decline from 10.00% in 2019 to 2.86% in 2023. In contrast, resistance rates for (nucleoside reverse transcriptase inhibitors)NRTIs and (non-nucleoside reverse transcriptase inhibitors)NNRTIs peaked in 2022 but subsequently declined to 10.00% and 27.14%, respectively, in 2023 (Fig. 1C). The overall drug resistance rate among elderly HIV-1/AIDS patients was at 37.98% (79/208) from 2018 to 2023. Drug resistance mutations (DRMs) were detected as follows: 4.33% (9/208) for PIs, 16.35% (34/208) for NRTIs, and 32.69% (68/208) for NNRTIs. As for the PI resistance, the most frequent mutations were Q58E/QE (2.40%, 5/208), which accounted for the highest proportion of low-level resistance to TPV/r (2.40%, 5/208). Regarding NRTIs resistance, M184V/I/MI/MV/MIV were linked to the highest levels of resistance to FTC and 3TC (13.94%, 29/208). Regarding NNRTIs resistance, NVP showed the highest level of high-level resistance (27.40%, 57/208). The most frequent mutations associated with NNRTIs resistance were V179D/E/T/VD (15.87%, 33/208) and K103N/KN/NS (15.38%, 32/208), as shown in Fig. 1D.

### Molecular transmission network

**Fig. 2:**
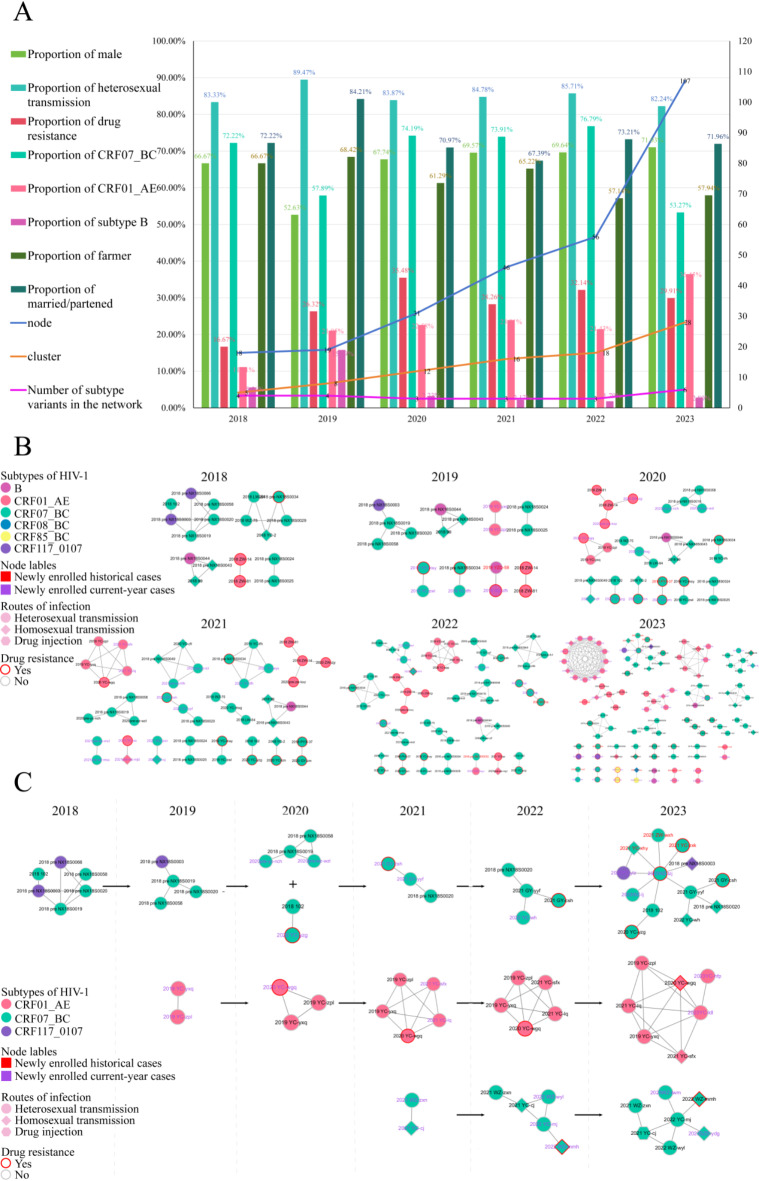
(**A**) Dynamic changes in key data of the molecular network. (**B**) Annual molecular transmission networks in elderly HIV/AIDS patients in NHAR from 2018 to 2023. (**C**) Dynamic evolution of three major transmission clusters.

By constructing and analyzing the molecular transmission networks of elderly HIV/AIDS patients in NHAR from 2018 to 2023, we observed significant network dynamics. Specifically, the number of nodes in the network increased significantly from 18 in 2018 to 107 in 2023. This growth was accompanied by a surge in molecular transmission clusters, which increased from 5 to 28, including the identification of 4 large-scale transmission clusters (nodes ≥ 7) in 2023 (Fig. 2B).

Analysis of the four large transmission clusters reveals that, except for the largest cluster, a newly identified transmission cluster in 2023, the remaining three clusters emerged or expanded from smaller clusters observed in previous years (Fig. 2C).

In terms of node characteristics, joined nodes were predominantly male, married/partnered, farmers, and infected through heterosexual transmission (Fig. 2A). The likelihood of males accessing the internet was 0.35 times that of females (AOR = 0.35, 95% CI: 0.152–0.820). The probabilities of individuals entering the network from Yinchuan, Shizuishan, and Wuzhong cities were 0.17 times (AOR = 0.17, 95% CI: 0.051–0.584), 0.17 times (AOR = 0.17, 95% CI: 0.04–0.79), and 0.23 times (AOR = 0.23, 95% CI: 0.06–0.83) that of Zhongwei city, respectively. In addition, the subtypes of network access decreased to 3 types from 2020 to 2022 and then rebounded to 6 types in 2023. CRF07_BC, CRF01_AE and subtype B are consistently represented, although CRF07_BC dominates and CRF01_AE shows an increasing trend. The probabilities of CRF07_BC and CRF01_AE subtypes entering the network are 3.00 times (AOR = 3.00, 95% CI: 1.26–7.16) and 8.08 times (AOR = 8.08, 95% CI: 2.96–22.04), respectively, those of other subtypes. HIV/AIDS patients with drug resistance were less likely to enter the network (AOR = 0.43, 95% CI: 0.24–0.80), as shown in Table 3. The network revealed connections between nodes reported within the same year and interconnections across different years. Notably, some previously unconnected case nodes from previous years that were initially excluded from the network were found to be connected to nodes from the current year, forming distinct cluster structures (Fig. 2B).

**Table 3:** The impact factors of the molecular transmission network in elderly HIV/AIDS patients in NHAR, 2018–2023.

Variable	Total (*n* = 208)	Enter network		One-factor logistic analysis		Multifactor logistic analysis	
		No (*n* = 101)	Yes (107)	COR (95% CI)	*P* value	AOR (95% CI)	*P* value
Age, (n/%)							
50 ~ 54	70 (33.65)	37 (36.63)	33 (30.84)	Ref.		Ref.	
55 ~ 59	46 (22.12)	24 (23.76)	22 (20.56)	1.03 (0.49–2.16)	0.943	1.07 (0.48–2.37)	0.867
60 ~ 64	34 (16.35)	15 (14.85)	19 (17.76)	1.42 (0.62–3.24)	0.404	1.59 (0.65–3.87)	0.306
65 ~ 69	20 (9.62)	9 (8.91)	11 (10.28)	1.37 (0.51–3.72)	0.536	1.84 (0.60–5.63)	0.282
70~	38 (18.27)	16 (15.84)	22 (20.56)	1.54 (0.69–3.42)	0.287	1.62 (0.66-4.00)	0.292
Gender, (n/%)							
Female	45 (21.63)	14 (13.86)	31 (28.97)	Ref.		Ref.	
Male	163 (78.37)	87 (86.14)	76 (71.03)	0.39 (0.20–0.80)	< 0.05	0.35 (0.15–0.81)	< 0.05
Occupation, (n/%)							
Unemployed/retiree	61 (29.33)	31 (30.69)	30 (28.04)	Ref.		Ref.	
Farmer	109 (52.40)	47 (46.54)	62 (57.94)	1.36 (0.73–2.56)	0.334	1.56 (0.77–3.18)	0.220
Business waiter	9 (4.33)	4 (3.96)	5 (4.67)	1.29 (0.32–5.28)	0.722	1.23 (0.28–5.41)	0.787
Workers	25 (12.02)	15 (14.85)	10 (9.35)	0.69 (0.28–1.77)	0.439	0.84 (0.29–2.46)	0.746
Others and unknown	4 (1.92)	4 (3.96)	0 (0.00)	-	-	-	-
Marital status, (n/%)							
Married/Partnered	140 (67.31)	64 (63.37)	76 (71.03)	Ref.		Ref.	
Divorced/Widowed	63 (30.29)	34 (33.66)	29 (27.10)	0.72 (0.40–1.30)	0.277	0.78 (0.41–1.47)	0.442
Unmarried	5 (2.40)	3 (2.97)	2 (1.87)	0.56 (0.09–3.46)	0.534	0.78 (0.11–5.27)	0.792
Educational level, (n/%)							
Primary school and Below	97 (46.63)	43 (42.57)	54 (50.47)	Ref.		Ref.	
Junior high school	74 (35.58)	38 (37.62)	36 (33.64)	0.75 (0.41–1.38)	0.363	1.10 (0.54–2.24)	0.797
High school/Technical secondary school	26 (12.50)	17 (16.83)	9 (8.41)	0.42 (0.17–1.04)		0.61 (0.22–1.67)	0.333
Junior college and above	11 (5.29)	3 (2.97)	8 (7.48)	2.12 (0.53–8.49)	0.287	2.91 (0.64–13.26)	0.168
Route of infection, (n/%)							
Heterosexual transmission	168 (80.77)	78 (77.23)	90 (84.11)	Ref.		Ref.	
Homosexual transmission	37 (17.79)	20 (19.80)	17 (15.89)	0.74 (0.36–1.50)	0.402	1.02 (0.46–2.23)	0.964
Drug injection	3 (1.44)	3 (2.97)	0 (0.00)	-	-		
Onset time, (n/%)							
2018~	27 (12.98)	7 (6.93)	20 (18.69)	Ref.		Ref.	
2019~	10 (4.81)	5 (4.95)	5 (4.67)	0.35 (0.08–1.58)	0.173	0.30 (0.05–1.56)	0.152
2020~	35 (16.83)	22 (21.78)	13 (12.15)	0.21 (0.07–0.62)	< 0.05	0.23 (0.07–0.73)	< 0.05
2021~	37 (17.79)	16 (15.84)	21 (19.63)	0.50 (0.16–1.35)	0.158	0.51 (0.16–1.59)	0.245
2022~	29 (13.94)	20 (19.80)	9 (8.41)	0.16 (0.05–0.51)	< 0.05	0.17 (0.05–0.59)	< 0.05
2023~	70 (33.65)	31 (30.69)	39 (36.45)	0.44 (0.17–1.17)	0.101	0.61 (0.21–1.75)	0.360
Drug resistance, (n/%)							
No	129 (62.02)	54 (53.47)	75 (70.09)	Ref.		Ref.	
Yes	79 (37.98)	47 (46.53)	32 (29.91)	0.49 (0.28–0.87)	< 0.05	0.43 (0.24–0.80)	< 0.05
Subtype							
Others^*^	40 (19.23)	29 (28.71)	11 (10.28)	Ref.		Ref.	
CRF01_AE	54 (25.96)	15 (14.85)	39 (36.45)	6.85 (2.75–17.11)	< 0.001	8.08 (2.96–22.04)	< 0.001
CRF07_BC	114 (54.81)	57 (56.44)	57 (53.27)	2.64 (1.20–5.78)	< 0.05	3.00 (1.26–7.16)	< 0.05
High school/Technical secondary school	26 (12.50)	17 (16.83)	9 (8.41)	0.42 (0.17–1.04)		0.61 (0.22–1.67)	0.333
Junior college and above	11 (5.29)	3 (2.97)	8 (7.48)	2.12 (0.53–8.49)	0.287	2.91 (0.64–13.26)	0.168
Route of infection, (n/%)							
Heterosexual transmission	168 (80.77)	78 (77.23)	90 (84.11)	Ref.		Ref.	
Homosexual transmission	37 (17.79)	20 (19.80)	17 (15.89)	0.74 (0.36–1.50)	0.402	1.02 (0.46–2.23)	0.964
Drug injection	3 (1.44)	3 (2.97)	0 (0.00)	-	-		
Onset time, (n/%)							
2018~	27 (12.98)	7 (6.93)	20 (18.69)	Ref.		Ref.	
2019~	10 (4.81)	5 (4.95)	5 (4.67)	0.35 (0.08–1.58)	0.173	0.30 (0.05–1.56)	0.152
2020~	35 (16.83)	22 (21.78)	13 (12.15)	0.21 (0.07–0.62)	< 0.05	0.23 (0.07–0.73)	< 0.05
2021~	37 (17.79)	16 (15.84)	21 (19.63)	0.50 (0.16–1.35)	0.158	0.51 (0.16–1.59)	0.245
2022~	29 (13.94)	20 (19.80)	9 (8.41)	0.16 (0.05–0.51)	< 0.05	0.17 (0.05–0.59)	< 0.05
2023~	70 (33.65)	31 (30.69)	39 (36.45)	0.44 (0.17–1.17)	0.101	0.61 (0.21–1.75)	0.360
Drug resistance, (n/%)							
No	129 (62.02)	54 (53.47)	75 (70.09)	Ref.		Ref.	
Yes	79 (37.98)	47 (46.53)	32 (29.91)	0.49 (0.28–0.87)	< 0.05	0.43 (0.24–0.80)	< 0.05
Subtype							
Others^*^	40 (19.23)	29 (28.71)	11 (10.28)	Ref.		Ref.	
CRF01_AE	54 (25.96)	15 (14.85)	39 (36.45)	6.85 (2.75–17.11)	< 0.001	8.08 (2.96–22.04)	< 0.001
CRF07_BC	114 (54.81)	57 (56.44)	57 (53.27)	2.64 (1.20–5.78)	< 0.05	3.00 (1.26–7.16)	< 0.05
Place of residence							
Zhongwei City	21 (10.10)	5 (4.95)	16 (14.95)	Ref.		Ref.	
Yinchuan City	106 (50.96)	55 (54.46)	51 (47.66)	0.29 (0.10–0.85)	< 0.05	0.17 (0.05–0.58)	< 0.05
Shizuishan City	17 (8.17)	10 (9.90)	7 (6.54)	0.22 (0.05–0.88)	< 0.05	0.17 (0.04–0.79)	< 0.05
Wuzhong City	44 (21.15)	23 (22.77)	21 (19.63)	0.29 (0.09–0.92)	< 0.05	0.23 (0.06–0.83)	< 0.05
Guyuan City	20 (9.62)	8 (7.92)	12 (11.21)	0.47 (0.12–1.80)	0.269	0.47 (0.11–2.04)	0.315

Others^*^ including B、C、CRF55_01B、CRF117_0107、CRF08_BC、CRF85_BC and URF subtype.

### Spatial analysis

Spatial autocorrelation analysis was performed to assess the annual and cumulative trends of elderly HIV/AIDS cases between 2018 and 2023. Notably, all global Moran’s I values were greater than 0(*P* < 0.05) from 2020 to 2023, indicating a highly significant clustering pattern of elderly HIV/AIDS patients across NHAR during these years (Table 4).

**Fig. 3:**
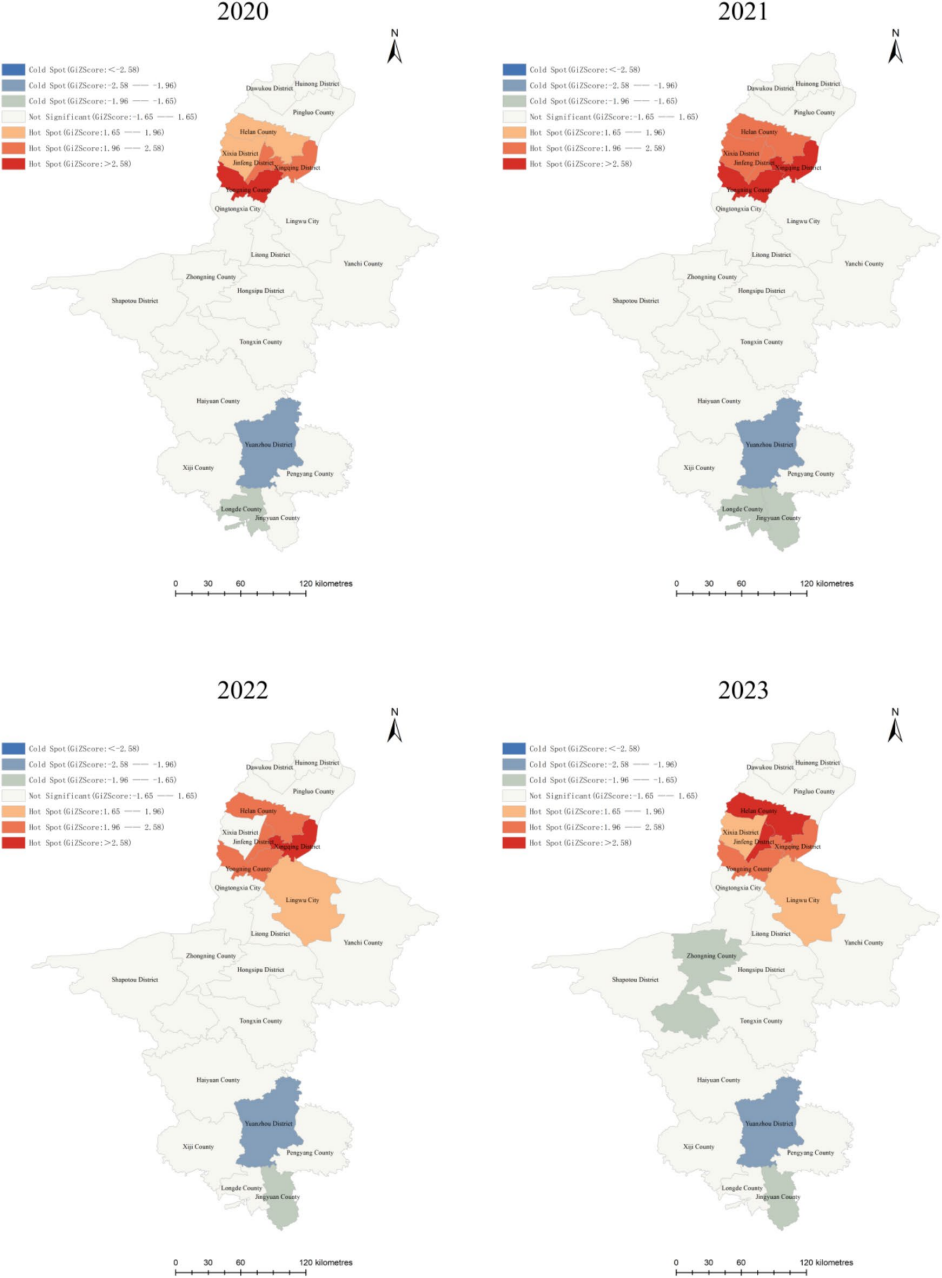
Analysis of spatial distribution hotspots of HIV/AIDS patients in NHAR from 2018 to 2023. (The base map is from the China National Bureau of Surveying, Mapping and Geoinformation, which provides a standard map download service, Review No. GS(2024)0650).

Local spatial autocorrelation was used to investigate the distribution of cold and hot spots at the county level within the NHAR region from 2020 to 2023. Between 2020 and 2021, hotspots expanded northwards from Yongning County and Xingqing District, and three new hotspots emerged in 2021: Jinfeng District, Xixia District, and Helan County, while the aggregation of the original hotspots intensified. In 2022, the hotspot areas began to shift southeast, with Lingwu City showing increased aggregation (90% Confidence). After briefly disappearing from the hotspot area, Xixia District showed renewed aggregation in 2023 (90% Confidence). Yuanzhou District remained a consistent cold spot from 2020 to 2023. From 2021 to 2023, there was a decline in the clustering of elderly HIV-1/AIDS patients in Jingyuan County (90% Confidence), and in 2023, Zhongning County also showed a similar trend of decreased clustering (90% Confidence) (Fig. 3).

**Table 4:** The general spatial autocorrelation of elderly HIV/AIDS patients in NHAR, 2018–2023.

Year	Moran’s I	Z-value	*P*-value
2018	0.149	1.341	0.180
2019	0.217	1.822	0.068
2020	0.296	2.351	0.019
2021	0.403	3.042	0.002
2022	0.359	2.780	0.005
2023	0.340	2.668	0.008

## Discussion

This study employs cross-sectional molecular epidemiological and spatial analytical techniques, providing the first comprehensive examination of the prevalence patterns and geographical distribution of HIV-1 infection among the elderly in NHAR.

The rate of HIV/AIDS infection among elderly males is significantly higher than that among females, with heterosexual transmission being the predominant mode, similar to the research findings in other regions of our country^13–15^. A plausible explanation for this disparity lies in the heightened sexual desire among males aged over 50, who are more prone to engage in non-marital heterosexual activities. Moreover, lower rates of condom use in this age group, often associated with factors such as erectile dysfunction in males and menopause in females, further exacerbate the spread of HIV-1^16^. Notably, a considerable proportion of the study participants were farmers with education levels of junior high school or below, exhibiting limited awareness of HIV transmission and prevention. Their frequent visits to low-cost sex venues and high mobility contribute to the inter-regional spread of HIV-1. Therefore, it is imperative to implement HIV-1 prevention initiatives and knowledge dissemination efforts in rural areas, targeting populations with low levels of education through multiple channels to increase their awareness and understanding of HIV.

In this study, CRF07_BC was the dominant subtype in both the heterosexual transmission population and the homosexual transmission population, which is consistent with that in the men who have sex with men population in Guangzhou and the men who have sex with men and heterosexual population in Guangxi^17,18^. In contrast to the results of a comprehensive nationwide molecular epidemiological study, which revealed CRF01_AE as the dominant strain in China after 2007^19^, the consistent predominance of CRF07_BC can be attributed to its rapid expansion within China’s sexually transmitted population in recent years. Additionally, the unique mutations in the P6 Gag protein of CRF07_BC, such as the PTAPPE (insPTAP) insertion and the PIDKELY (30PIDKELY36) deletion, have significantly affected the invasion and replication capabilities of the virus. These mutations contribute to slower disease progression, reduced virulence, and enhanced transmissibility of CRF07_BC, ultimately leading to longer survival times for infected individuals^20,21^. It is therefore imperative to strengthen comprehensive treatment, care and rigorous monitoring of disease progression in older people living with HIV.

During the study period from 2018 to 2023, we observed a marked increase in the diversity of HIV-1 subtypes, incorporating not only the consistently low-prevalence subtype B, but also the emerging second-generation recombinant subtype CRF117_0107 and the regionally identified CRF85_BC in 2023. This underscores the profound increase in viral diversity and the escalating intricacies of the epidemic. Therefore, a rigorous, science-based approach to epidemic prevention and control is essential, focusing on strengthening surveillance, research, public awareness and adapting strategies to the evolving virus.

The pronounced variability of HIV-1 presents another formidable challenge in the form of drug resistance, emerging as a significant impediment to epidemic control efforts. Our study in NHAR showed that the overall drug resistance prevalence was 37.98%, which exceeded the WHO alert threshold for moderate HIV-1 resistance (5-15%)^22^. It was higher than the HIV drug resistance rate in China from 2018 to 2023 and the HIV drug resistance rate in the population of Guangxi Zhuang Autonomous Region^17,23^. Two main factors contribute to this situation: First, as the immune system function gradually declines in the elderly, the HIV-1 virus is more likely to mutate within the body to evade the immune response, facilitating the emergence of drug resistance. Second, elderly patients tend to have multiple comorbidities and require concomitant medications, which increases the likelihood of pharmacological interactions that can affect the absorption, metabolism, and excretion of drugs, ultimately elevating the risk of drug resistance. The resistance rate observed in this subpopulation is significantly higher than the overall resistance rate in the region^24,25^, likely due to the delayed detection of resistance in the elderly population. This underscores the importance of strengthening HIV-1 surveillance in this vulnerable group to better understand and manage these challenges^26^. Therefore, it is necessary to strengthen surveillance efforts specifically targeting elderly patients and to develop tailored testing and treatment standards for them.

Males were significantly less likely to join the network than females. This may be due to the increased vulnerability of older women in sexual transmission scenarios. Physiologically, females have a greater exposure area and age-related changes such as vaginal thinning and dryness, which can facilitate vaginal wall trauma, increasing susceptibility to HIV-1^26^. Socially, unprotected sex with multiple partners or relationships with HIV-1 infected individuals puts females at greater risk of participating in the HIV-1 transmission chain. Additionally, gender inequality in underdeveloped regions often positions females at a disadvantage in sexual relationships, making it difficult for them to refuse unsafe sex or to request protection. This inequality exacerbates the risk of HIV-1 infection among females.

Interestingly, while nodes from Zhongwei city are fewer than those from Yinchuan and Shizuishan, the probability of entering the network is higher. This could be due to a smaller social network and lower population mobility in Zhongwei, suggesting that infections occurring here may have more traceable transmission routes.

Among the HIV-1 subtypes, CRF01_AE has the highest probability of entering the network, with a generally high node degree value. The largest transmission cluster identified in 2023 comprises CRF01_AE, displaying tight node connections. This may be because when CRF01_AE spreads, it can serve as a recombination backbone and carry specific gene fragments, such as drug resistance sites. These sites can enhance the virus’s drug resistance and replication ability, making the new subtype more likely to infect new hosts or survive longer in body fluids, thereby increasing the chance of transmission throughout the population^27,28^. Studies have shown that CRF01_AE can act as a recombination skeleton during transmission and carry specific gene fragments related to drug resistance^26^. These features have been suggested to enhance the virus’s drug resistance and replication ability, allowing it to survive longer in body fluids, which may increase transmission opportunities and the likelihood of new subtypes emerging. Furthermore, due to its high proportion of X4 tropism^27,28^, CRF01_AE is often associated with rapid disease progression and advanced immune deficiency^29^. Even with combination ART, poor immune recovery is commonly observed in CRF01_AE infections^30^. Therefore, it is essential to remain vigilant against the rapid spread of the newly discovered CRF01_AE transmission cluster and to take measures to prevent its expansion.

In our comprehensive analysis of HIV-1 transmission dynamics, we identified the emergence of four significant transmission clusters in 2023. Notably, three of these clusters illustrate a concerning trend of consolidation and expansion originating from smaller, previously established clusters. This finding underscores the dynamic and interconnected nature of HIV-1 epidemiology, wherein even modest outbreaks can evolve into more substantial transmission networks over time. Identifying these large transmission clusters, particularly those resulting from the consolidation of smaller clusters, provides valuable insights into the underlying mechanisms of HIV-1 transmission and may inform the development of more effective prevention and control strategies. It also emphasises the need for sustained genetic surveillance and sequencing efforts, which are essential to uncover hidden transmission routes and improve our understanding of the evolving epidemiology of HIV-1.

Spatial analysis from 2020 to 2023 revealed a significant spatial correlation among elderly HIV/AIDS patients. Local spatial autocorrelation analysis identified consistent cold spot characteristics in the districts and counties administered by Guyuan City. This observation may be attributed to the mountainous terrain and inconvenient transport networks in the southern region of Guyuan City, which are situated amidst rugged hills that may have acted as barriers to the spread of HIV-1, subsequently limiting viral circulation in the different areas. Secondly, as an ethnic minority community primarily consisting of the Hui ethnicity, the relatively conservative cultural mindset of the locals may influence their health beliefs, sexual education, and understanding of HIV-1 prevention and control, which could be a significant factor contributing to the formation of these cold spots.

Hot spots were identified in the districts and counties under the jurisdiction of Yinchuan City between 2020 and 2023, likely due to its advanced medical resources as NHAR’s capital city. Additionally, the relatively developed economy of the Yinchuan region has made it a significant destination for population migration from surrounding areas, resulting in increased population density, particularly among migrant laborers. This phenomenon of population mobility has broadened the transmission routes of HIV-1, potentially accelerating the spread of the virus across different regions and populations. The hotspot expanded northwards from Yongning and Xingqing districts, and Jinfeng, Xixia districts, and Helan county were added as hotspots in 2021 from 2020 to 2021. This may indicate that the source of infection is spreading outwards from the original hotspot area and the transmission pathway is expanding northwards. There may have been some high-risk behavioural patterns or population mobility factors that contributed to the spread of the virus from the original hotspot to the new area. The hotspot area shifted to the southeast in 2022, with increased clustering in Lingwu City, further suggesting that the source of infection may have shifted in response to changes in population mobility, economic activity, or patterns of social interaction. It is possible that increased economic activity in Lingwu City during this period attracted a large influx of people, some of whom may have been infected, leading to increased virus transmission in the region. The fact that Xixia District showed clustering again in 2023 after briefly moving out of the hotspot range may indicate that there is a potential source of ongoing infection in the region, where transmission may have previously been temporarily suppressed due to prevention and control measures or other factors, but changes in certain factors have reactivated the transmission chain. Free and anonymous HIV testing has been increased in community health centres, hospitals, and high-risk places to facilitate the early detection of infected persons. Health education on AIDS prevention has been strengthened, especially for participants in high-risk social venues. Previous preventive measures have been evaluated. Strategies have been adjusted in the light of the evaluation results, and a more rigorous continuous monitoring system has been implemented to detect and intervene in potential transmission risks.

There are limitations to this study. Firstly, the data analyzed in this study exclusively encompasses HIV-1 infected individuals within NHAR, potentially introducing bias into the spatial autocorrelation analysis due to cross-regional mobility patterns and data boundary effects. Secondly, a relatively large proportion of patients were excluded from the study due to a lack of sequencing or demographic data, which can lead to selection bias. Future studies should optimize sample collection strategies to enhance the representativeness and consistency of samples.

## Conclusions

In summary, this study demonstrates the innovative integration of interdisciplinary approaches, including epidemiology and spatial statistics, to facilitate precision public health interventions. At the epidemiological level, recent trends in HIV-1/AIDS among the elderly population in NHAR reveal a preponderance of male patients with low educational attainment and a predominantly agricultural background, with heterosexual transmission being the primary mode of infection. This population exhibits increased subtype diversity, accompanied by high levels of drug resistance and many resistance sites, with CRF01_AE and CRF07_BC emerging as the dominant circulating subtypes in recent years. The network has observed the expansion of transmission clusters and the emergence of large-scale clusters over the years. The transmission patterns are complex and characterized by drug resistant transmission and cross-city transmission phenomena. CRF01_AE and CRF07_BC pose significant transmission risks. Therefore, establishing a long-term dynamic monitoring molecular transmission network is particularly important for implementing targeted intervention measures. In terms of spatial distribution, the elderly HIV-1/AIDS patients in NHAR exhibit a pronounced clustering pattern, with the southern mountainous regions serving as cold spots for HIV-1 infection and distribution. In contrast, districts under the jurisdiction of Yinchuan City show a high density of cases. To effectively curb the spread of HIV-1 among the elderly, several strategic measures are essential: Firstly, it is critical to develop targeted testing and treatment guidelines and to strengthen surveillance initiatives for the older patient population. Secondly, constructing a long-term dynamic monitoring molecular transmission network provides a foundation for scientific identification and intervening in complex transmission patterns in the region. Lastly, it is imperative to fully mobilize medical resources, increase access to HIV-1 testing, put in place efficient screening procedures to promptly detect possible infections, and fortify the oversight and management of subsequent treatment.

## Data Availability

The datasets analyzed during the current study are not publicly available, but are available from the corresponding author on reasonable request.

## Abbreviations

HIV: Human immunodeficiency virus
ART: Antiretroviral therapies
NHAR: Ningxia Hui autonomous region
HIV-1: Human immunodeficiency virus type 1
